# Association between vitamin D receptor *BsmI, FokI*, and *Cdx2* polymorphisms and osteoporosis risk: an updated meta-analysis

**DOI:** 10.1042/BSR20201200

**Published:** 2020-07-15

**Authors:** Bin Chen, Wang-fa Zhu, Yi-yang Mu, Biao Liu, Hong-zhuo Li, Xiao-feng He

**Affiliations:** 1 Changzhi Medical College, No. 161, Jiefangdong Street, Shanxi Province, Changzhi 046000, China; 2 Department of Orthopaedics, Heping Hospital Affiliated to Changzhi Medical College, Shanxi, Changzhi 046000, China; 3 Department of Science and Education, Heping Hospital Affiliated to Changzhi Medical College, Shanxi, Changzhi 046000, China

**Keywords:** meta-analysis, osteoporosis, polymorphism, risk, VDR

## Abstract

**Background:** Many studies have reported the association between vitamin D receptor (*VDR*) polymorphism and osteoporosis risk. However, their results were conflicting. Six previous meta-analyses have been published to analyze *VDR* BsmI, FokI, and Cdx2 polymorphisms on osteoporosis risk. However, they did not evaluate the reliability of statistically significant associations. Furthermore, a lot of new articles have been published on these themes, and therefore an updated meta-analysis was performed to further explore these issues.

**Objectives:** To explore the association between *VDR* BsmI, FokI, and Cdx2 polymorphisms polymorphisms and osteoporosis risk.

**Methods:** The odds ratios (ORs) and 95% confidence intervals (95% CIs) were pooled to evaluate the association between *VDR BsmI, FokI*, and *Cdx2* polymorphisms and osteoporosis risk. To evaluate the credibility of statistically significant associations, we applied the false-positive report probabilities (FPRPs) test and the Venice criteria.

**Results:** Overall, statistically significantly increased osteoporosis risk was found in Indians and women for *VDR* FokI polymorphism. Statistically significantly decreased osteoporosis risk was found in West Asians for *VDR* BsmI polymorphism. However, when we performed a sensitivity analysis after excluding low quality and Hardy–Weinberg Disequilibrium (HWD) studies, significantly decreased osteoporosis risk was only found in overall population for *VDR* BsmI polymorphism. Further, less-credible positive results were identified when we evaluated the credibility of positive results.

**Conclusion:** These positive findings should be interpreted with caution and indicate that significant association may most likely result from less-credible, rather than from true associations or biological factors on the VDR *BsmI* and *FokI* polymorphisms with osteoporosis risk*.*

## Introduction

Osteoporosis is a systemic skeletal disease characterized by a systemic impairment of bone mass and microarchitecture that results in a high risk of fractures [1]. According to WHO, osteoporosis is the reduction in bone density below 2.5 standard deviation from the average for healthy and mature adults with similar ethnicity and age. It is one of the most common metabolic bone diseases in the world, affecting women over the age of 59 and men over the age of 74 [2]. It was reported that there were approximately 200 million osteoporosis patients in the world [3]. Therefore, it is very important to explore the potential pathogenic factors.

Multiple factors were reported to affect osteoporosis, including environmental factors such as exercise, smoking and alcohol consumption, metabolic syndrome, and genetic factors [4–6]. Among them, genes were a very important factor. The heritability of osteoporosis-related traits (such as bone mineral density) was reported to be up to 60–80% [7]. Up till now, tens of hundreds of risk genes have been identified for osteoporosis, including collagen type I α 1 gene (COL1A1), calcitonin receptor (CTR), estrogen receptor (ESR), vitamin D receptor (VDR), and so on [8–10]. Most of these genes are known to influence the reabsorption of bone by osteoclasts and the formation of bone by osteoblasts.

VDR was the most extensively reported, located on chromosome 12q13 [11], through mediating 1,25-dihydroxycholecalciferol (1,25(OH)_2_D_3_) to play a variety of biological effects [12]. In human monocytes, 1,25(OH)_2_D_3_ modulates chromatin accessibility at 8979 loci [13]. Therefore, VDR polymorphisms were associated with a variety of diseases, including bone mineral density and osteoporosis [14,15]. Morrison et al. [16] first investigated that variability in osteocalcin levels reflect allelic variation in the VDR gene. Since then, a large number of studies have reported that VDR gene mutations (such as *FokI* (rs10735810), *BsmI* (rs1544410) and *Cdx2* (rs11568820) were related to osteoporosis risk. However, these results were inconsistent or even conflicting. For example, Ling et al. [15] found that *VDR* Cdx-2 A allele was associated with decreased bone mineral density (BMD) risk and increased fracture risk. On the contrary, A allele was found to have protective effect on osteoporotic fractures in some studies [14,17]. Similarly, they were also conflicting in different studies [18–23] on the associations between the *VDR* FokI and BsmI polymorphisms and osteoporosis risk. These different results may be caused by small sample size, different races, regions, and sampling methods. Although several related meta-analyses have reported the associations between *VDR* BsmI, FokI, and Cdx2 polymorphisms and the risk of osteoporosis [24–29]. However, their studies have some disadvantages. First, the results of these meta-analyses were inconsistent. For example, Jia et al. [27] found that the *VDR* BsmI polymorphism may have a protective effect on the development of osteoporosis. However, Gang et al. [28] concluded that there was no association between *VDR* BsmI polymorphism and osteoporosis risk. Second, literature quality assessments had not been performed in some studies [24,25,27–29]. In addition, they did not evaluate the credibility of statistically significant associations [24–29]. Furthermore, some new studies have been published on the VDR polymorphisms and osteoporosis risk. Therefore, we performed an updated meta-analysis to provide more reliable results on these issues.

## Materials and methods

### Search strategy

We performed the meta-analysis according to the guidelines of the Preferred Reporting Items for Systematic Review and Meta-Analysis (PRISMA) group [30]. Databases including PubMed, Embase, and Chinese Wanfang Data Knowledge Service Platform were searched to investigate the association between VDR polymorphisms and osteoporosis risk. The following search strategy were used: (VDR OR vitamin D receptor OR BsmI OR FokI OR Cdx2) AND (polymorphism OR mutaion OR variant) AND (osteoporosis OR osteoporoses). The search deadline was November 2019.

### Selection criteria

The inclusion criteria were as follows: (1) case–control or cohort studies; (2) describe the association among *VDR* BsmI, FokI, and Cdx2 polymorphisms and osteoporosis risk; (3) the case and control groups have sufficient genotype data in the selected literature.

The exclusion criteria were: (1) duplicated studies; (2) studies without available data; (3) case reports, reviews, letters, and meta-analyses.

### Data extraction

The data extraction tables in the present study were prepared in advance. According to the established inclusion and exclusion criteria, the data were independently extracted and cross-checked; if there was any objection, the consensus can not be reached after discussion and negotiation. The third author was invited to extract the data again, and finally check and confirm. If the data are not detailed or in doubt, try to contact the original author, supplement and confirm the accuracy and integrity of the data. The extracted information was as follows: first author’s surname, publication year, country, ethnicity, age of cases and controls, the number of cases and controls, diagnostic criteria for osteoporosis, menopausal status, matching variables, site of BMD measurement, and number of genotype distributions in cases and controls.

### Quality assessment

The quality of all eligible studies was independently assessed by the two authors. We designed quality assessment criteria on the basis of two previous meta-analyses [31,32]. Supplementary Table S1 lists the scale for quality assessment of molecular association studies of osteoporosis risk. The total score was 20 points, studies scoring above 12 were excellent, those scoring less than 9 were poor, and those scoring between 9 and 12 were moderate.

### Statistical analysis

The odds ratios (ORs) and 95% confidence intervals (95% CIs) were pooled to evaluate the association strength, *P*<0.05 was considered as statistically significant. Five genetic model comparisons were used: (1) allele model; (2) additive model; (3) dominant model; (4) recessive model; (5) overdominant model. Heterogeneity test used Chi-square-based Q-test and *I^2^* test. There was no obvious heterogeneity among studies when *P*>0.10 and/or *I^2^* ≤ 50% [33] and the ORs were pooled to apply a fixed-effects model [34]. Otherwise, a random-effects model was selected [35]. Furthermore, a meta-regression analysis was applied to explore sources of heterogeneity. Subgroup analyses were performed according to ethnicity or gender. Sensitivity analysis was estimated by the following three methods: (1) a single study was removed each time; (2) exclude low quality and Hardy–Weinberg Disequilibrium (HWD) studies; (3) the studies met the following conditions: high-quality studies, Hardy–Weinberg Equilibrium (HWE), and matching studies. Chi-square goodness-of-fit test was applied to examine HWE, and it was considered as HWE in control groups if *P*>0.05. In addition, the false-positive report probabilities (FPRP) test [36] and the Venice criteria [37] were applied to assess the credibility of statistically significant associations. Begg’s funnel plot [38] and Egger’s test were used to evaluate the publication bias [39]. All statistical analyses were conducted using Stata 12.0 software.

## Results

### Description of included studies

**Figure 1:**
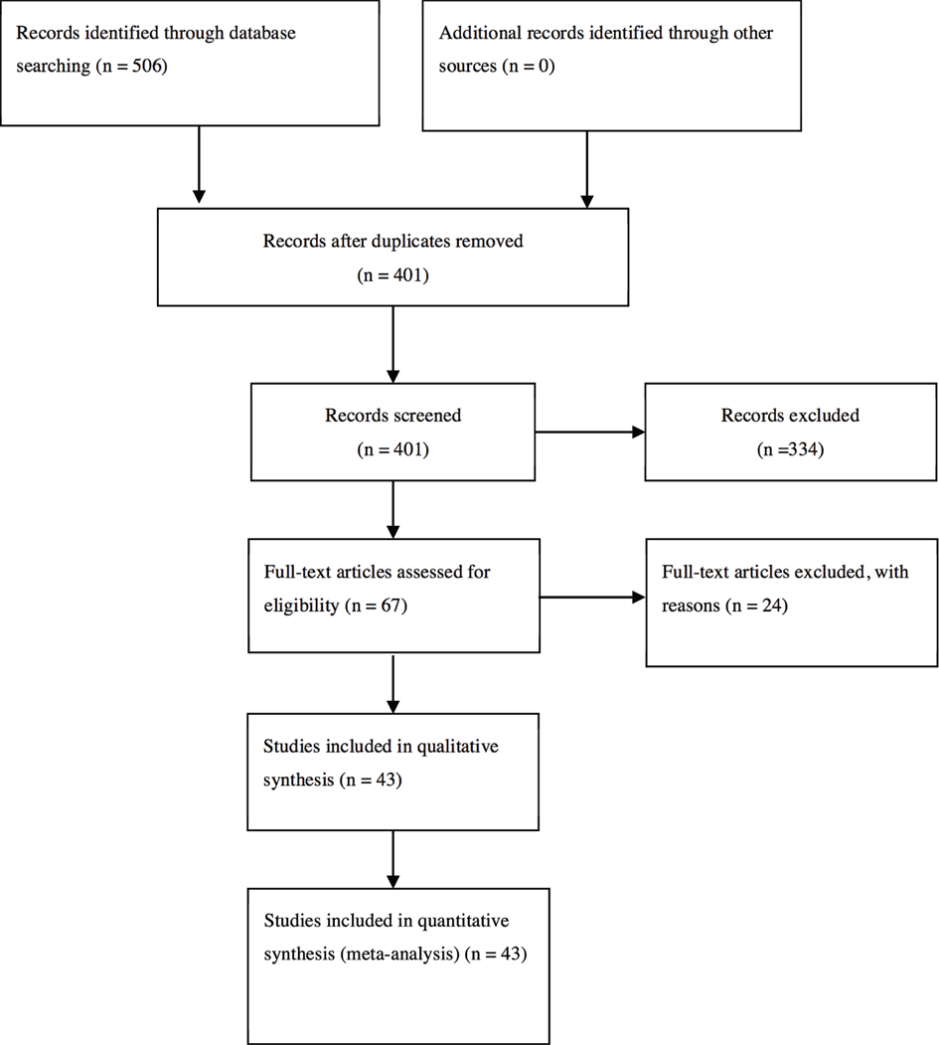
Flow diagram of the literature search

We got 506 articles by searching, according to the inclusion and exclusion criteria, 43 studies met our requirements (involving 4680 osteoporosis cases and 5373 controls) [21,22,40–80], of which 34 studies explored the association between *VDR* BsmI and osteoporosis risk (involving 2973 osteoporosis cases and 3724 controls), 19 studies reported *VDR* FokI (involving 3694 osteoporosis cases and 2943 controls), and 4 studies explored *VDR* Cdx2 (involving 378 osteoporosis cases and 743 controls). In addition, 23, 11, 4, 3, 1, and 1 case–control studies were conducted to analyze Caucasians, East Asians, West Asians, Indians, Southeast Asians, and Africans, respectively. Among them, seven studies were performed to examine the association between men and osteoporosis risk, and 38 studies explored the association between women and osteoporosis risk. Thirty studies on postmenopausal women, two studies on premenopausal women, and nine studies did not describe menopause status. Finally, there were 9 high-quality studies, 20 medium-quality studies, and 5 low-quality studies on *VDR* BsmI; 7 high-quality studies, 10 medium-quality studies, and 2 low-quality studies on *VDR* FokI; and 3 medium-quality studies and 1 low-quality study on *VDR* Cdx2. The detailed characteristics and scoring of each study are displayed in Table 1. The literature selection and inclusion processes are shown in Figure 1. The genotype frequencies of *VDR* BsmI, FokI, and Cdx2 polymorphisms with osteoporosis risk and HWE test results were shown in Tables 2–4.

**Table 1:** Main characteristics and quality score of studies included

First author/year	Country	Ethnicity	Gender	Cases						Controls					Score
				*n*	Age^1^	Menopause	BMD site	Diagnosis	Matching	*n*	Healthy	Age^1^	Menopause	BMD site	
Kow, 2019	British	Caucasian	Men	69	58.96 ± 12.78	Ne	LS-fn	WHO	Age and Sex	121	Yes	64.98 ± 10.06	Ne	LS-hip	15
Techapatiphandee, 2018	Thai	Southeast Asian	Female	105	73.10 ± 8.90	PSM	LS-hip	WHO	Sex	132	Yes	63.40 ± 8.70	PSM	LS-hip	13
Ahmad, 2018	India	Indian	Female	254	56.12 ± 7.00	PSM	LS-hip-fn	WHO	Age and Sex	254	Yes	55.11 ± 5.66	PSM	LS-hip	14
Meng, 2017	China	East Asian	Female	90	67.20 ± 8.60	Ne	LS-hip	Ne	Sex	246	Yes	55.90 ± 9.60	Female	LS-hip	8
Dehghan, 2016	Iran	West Asian	Men	130	46.10 ± 6.00	Ne	LS-fn	WHO	Sex	70	Yes	46.10 ± 6.00	Men	LS-hip	10
Ziablitsev, 2015	Ukraine	Caucasian	Female	30	Ne	PSM	Ne	Ne	Sex	44	Yes	Ne	PSM	Ne	8
Mohammadi, 2015	Iran	West Asian	Female	142	58.10 ± 7.90	PSM	LS-hip-fn	WHO	Age and Sex	31	Yes	58.10 ± 7.90	PSM	LS-hip-fn	14
Mohammadi, 2015	Iran	West Asian	Female	101	35.40 ± 9.00	Pre	LS-hip-fn	WHO	Age and Sex	374	Yes	35.40 ± 9.00	Pre	LS-hip-fn	15
Mohammadi, 2015	Iran	West Asian	Men < 50	75	32.90 ± 8.60	Ne	LS-hip-fn	WHO	Age and Sex	195	Yes	32.90 ± 8.60	Ne	LS-hip-fn	15
Mohammadi, 2015	Iran	West Asian	Men ≥ 50	112	61.20 ± 8.90	Ne	LS-hip-fn	WHO	Age and Sex	24	Yes	61.20 ± 8.90	Ne	LS-hip-fn	14
Moran, 2015	Spanish	Caucasian	Female	150	60.24 ± 7.74	PSM	LS-fn	WHO	Age and Sex	30	Yes	59.73 ± 9.28	PSM	LS-fn	16
Boroń, 2015	Poland	Caucasian	Female	278	Ne	PSM	LS	Ne	Age and Sex	292	Yes	Ne	PSM	LS	13
Marozik, 2013	Belarus	Caucasian	Female	54	58.30 ± 6.20	PSM	LS-fn	WHO	Age and BMI	77	Yes	56.70 ± 7.40	PSM	LS-fn	11
González, 2013	Mexico	Caucasian	Female	88	57.65 ± 5.58	PSM	LS-fn	WHO	Sex	88	Yes	56.34 ± 4.98	PSM	LS-fn	11
Pouresmaeili, 2013	Iran	West Asian	Female	64	53.53 ± 9.80	Ne	LS-fn	WHO	Age and Sex	82	Yes	53.53 ± 9.80	Ne	LS-fn	12
Efesoy, 2011	Turkey	Caucasian	Female	40	65.75 ± 9.80	PSM	LS-fn	WHO	Sex	30	Yes	62.40 ± 8.70	PSM	LS-fn	11
Yasovanthi, 2011	India	Indian	Female	247	57.70 ± 4.60	PSM	LS	WHO	Age and Sex	254	Yes	57.70 ± 4.60	PSM	LS	16
Yasovanthi, 2011	India	Indian	Female	180	39.50 ± 4.40	Pre	LS	WHO	Age and Sex	206	Yes	39.50 ± 4.40	Pre	LS	15
Xing, 2011	China	East Asian	Female	32	72.50 ± 6.40	Ne	LS	T-score < 2.0	Sex	70	Yes	70.50 ± 5.20	Female	LS	9
Mansour, 2010	Egypt	African	Female	50	54.40 ± 5.10	PSM	LS-fn	WHO	Age and Sex	20	Yes	53.50 ± 5.40	PSM	LS-fn	8
Durusu, 2010	Turkey	Caucasian	Female	50	58.30 ± 6.50	PSM	LS-hip-fn	WHO	Sex	50	Yes	57.30 ± 6.60	PSM	LS-hip-fn	11
Gu, 2010	China	East Asian	Female	33	58.40 ± 6.30	PSM	Fn	WHO	Sex	148	Yes	58.40 ± 6.30	PSM	Fn	11
Gu, 2010	China	East Asian	Men	8	61.60 ± 7.00	Ne	Fn	WHO	Sex	260	Yes	61.60 ± 7.00	Men	Fn	12
Mencej, 2009	Slovenia	Caucasian	Female	239	64.50 ± 8.20	PSM	LS-hip-fn	WHO	Sex	228	Yes	61.50 ± 8.30	PSM	LS-hip-fn	12
Seremak, 2009	Poland	Caucasian	Female	163	64.27 ± 8.72	PSM	LS	WHO	Sex	63	Yes	63.08 ± 7.24	PSM	LS	10
Uysal, 2008	Turkey	Caucasian	Female	100	Ne	PSM	LS-fn	WHO	Sex	146	Yes	Ne	PSM	LS-fn	12
Pérez, 2008	Argentina	Caucasian	Female	64	62.70 ± 0.86	PSM	LS-fn	WHO	Sex	68	Yes	59.40 ± 0.85	PSM	LS-fn	14
Mitra, 2006	India	Indian	Female	119	54.2 ± 3.40	PSM	LS-fn	WHO	Sex	97	Yes	54.20 ± 3.40	PSM	LS-fn	11
Zhang, 2006	China	East Asian	Men	26	70.5 ± 5.30	Ne	LS	T-score < 2.0	Sex	66	Yes	73.40 ± 4.30	Men	LS	7
Liu, 2005	China	East Asian	Men	89	Ne	Ne	LS-hip	T-score < 2.0	Sex	56	Yes	Ne	Men	LS-hip	10
Zhu, 2004	China	East Asian	Female	40	Ne	PSM	LS-fn	WHO	Sex	158	Yes	Ne	PSM	LS-fn	10
Duman, 2004	Turkey	Caucasian	Female	75	53.16 ± 1.31	PSM	LS-hip	WHO	Age and Sex	66	Yes	52.62 ± 1.69	PSM	LS-hip	10
Lisker, 2003	Mexico	Caucasian	Female	65	65.20 ± 6.80	PSM	LS-fn	WHO	Sex	57	Yes	56.50 ± 6.00	PSM	LS-fn	11
Douroudis, 2003	Greece	Caucasian	Female	35	61.37 ± 0.96	PSM	Forearm	WHO	Sex	44	Yes	58.68 ± 1.01	PSM	Forearm	12
Chen, 2003	China	East Asian	Female	78	54.72 ± 2.60	PSM	Forearm	T-Score < 2.0	Sex	81	Yes	53.68 ± 2.90	PSM	Forearm	9
Zajickova, 2002	Czech	Caucasian	Female	65	60.10 ± 10.30	PSM	LS-hip	WHO	Sex	33	Yes	63.60 ± 7.80	PSM	LS-hip	10
Pollak, 2001	Israel	West Asian	Female	75	Ne	Ne	LS-fn	WHO	Sex	143	Yes	Ne	Ne	LS-fn	13
Langdahl, 2000	Aarhus	Caucasian	Men	30	55.70 ± 11.00	Ne	LS-hip	WHO	Age and Sex	73	Yes	51.10 ± 15.70	Ne	LS-hip	13
Langdahl, 2000	Aarhus	Caucasian	Female	80	58.20 ± 6.40	Ne	LS-hip	WHO	Age and Sex	80	Yes	56.20 ± 7.70	Ne	LS-hip	13
Fontova Garrofe, 2000	Spanish	Caucasian	Female	75	58.30 ± 5.00	PSM	LS-hip	WHO	Sex	51	Yes	57.20 ± 4.50	PSM	LS-hip	9
Choi, 2000	Korea	East Asian	Female	48	55.10 ± 6.00	PSM	LS-fn	WHO	Sex	65	Yes	55.10 ± 6.00	PSM	LS-fn	11
Zhang, 1998	China	East Asian	Female	17	56. 76	Ne	LS	Ne	Sex	52	Yes	54.38	Female	LS	6
Lucotte, 1999	French	Caucasian	Female	124	63.00 ± 12.30	PSM	LS-fn	WHO	Age and Sex	105	Yes	63.00 ± 12.30	PSM	LS-fn	15
Gennari, 1999	Italian	Caucasian	Female	164	57.70 ± 0.60	PSM	LS	WHO	Sex	119	Yes	56.90 ± 0.60	PSM	LS	12
Gennari, 1998	Italian	Caucasian	Female	155	58.20 ± 0.60	PSM	LS	WHO	Sex	136	Yes	57.10 ± 0.70	PSM	LS	12
Vandevyver, 1997	Belgium	Caucasian	Female	698	75.20 ± 4.70	PSM	LS-fn	Ne	Sex	86	Yes	66.30 ± 8.40	PSM	LS-fn	9
Tamai, 1997	Japan	East Asian	Female	90	71.00 ± 10.00	Ne	LS	Ne	Sex	92	Yes	43.00 ± 17.00	Female	LS	7
Yanagi, 1996	Japan	East Asian	Female	23	Ne	Ne	LS	Ne	Sex	66	Yes	Ne	Female	LS	7
Houston, 1996	U.K.	Caucasian	Female	44	66.00 ± 0.85	Ne	LS-hip	WHO	Sex	44	Yes	65.30 ± 0.95	Female	LS-hip	13

Abbreviations: Fn, femoral neck; LS, lumbar spine; N, not available; Pre, premenopause; PSM, postmenopausal. ^1^Mean ± SD years.

**Table 2:** Genotype frequencies of VDR *BsmI* polymorphism in studies included in this meta-analysis

First author/year	Ethnicity	Gender	Case			Control			HWE	
			BB	Bb	bb	BB	Bb	bb	Chi-square test	*P*
Kow, 2019	Caucasian	Male	31	66	21	11	34	13	1.752	0.1856
Techapatiphandee, 2018	Southeast Asian	Female	85	19	1	103	25	4	2.377	0.1231
Ahmad, 2018	Indian	Female	54	137	63	54	152	48	9.909	0.0016
Meng, 2017	East Asian	Female	4	12	74	6	24	216	19.383	0
Dehghan, 2016	West Asian	Male	31	70	29	14	39	17	0.947	0.3304
Moran, 2015	Caucasian	Female	18	65	67	3	19	8	2.752	0.0972
Boroń, 2015	Caucasian	Female	101	121	56	128	113	51	8.26	0.0041
Marozik, 2013	Caucasian	Female	12	31	11	11	26	40	3.495	0.0616
González-Mercado, 2013	Caucasian	Female	6	28	54	4	38	46	1.234	0.2667
Pouresmaeili, 2013	West Asian	Female	14	33	17	13	33	36	1.31	0.2524
Efesoy, 2011	Caucasian	Female	5	23	12	5	15	10	0.024	0.8756
Mansour, 2010	African	Female	27	15	8	1	2	17	3.951	0.0469
Mencej-Bedrac, 2009	Caucasian	Female	27	110	103	40	100	88	1.538	0.2149
Seremak, 2009	Caucasian	Female	27	66	70	10	27	26	0.442	0.5062
Durusu, 2010	Caucasian	Female	15	19	16	19	7	24	25.717	0
Uysal, 2008	Caucasian	Female	18	48	34	24	78	44	1.155	0.2826
Pérez, 2008	Caucasian	Female	17	35	12	20	32	16	0.21	0.6469
Mitra, 2006	Indian	Female	51	46	22	19	38	40	3.072	0.0796
Liu, 2005	East Asian	Male	2	11	76	0	6	50	0.179	0.6719
Zhu, 2004	East Asian	Female	6	26	8	7	105	46	27.257	0
Duman, 2004	Caucasian	Female	18	54	3	24	72	4	25	0
Lisker, 2003	Caucasian	Female	15	17	34	13	38	6	7.133	0.0076
Douroudis, 2003	Caucasian	Female	3	12	20	10	29	5	4.95	0.0261
Chen, 2003	East Asian	Female	0	13	65	0	12	69	0.518	0.4715
Zajickova, 2002	Caucasian	Female	21	24	20	10	13	10	1.485	0.223
Pollak, 2001	West Asian	Female	18	50	32	11	47	42	0.16	0.6896
Langdahl, 2000	Caucasian	Male	8	16	6	15	28	30	2.893	0.089
Langdahl, 2000	Caucasian	Female	23	38	19	25	34	21	1.749	0.186
Fontova, 2000	Caucasian	Female	9	49	17	10	22	19	0.612	0.4341
Zhang, 1998	East Asian	Female	0	3	14	0	3	49	0.046	0.8304
Gennari, 1998	Caucasian	Female	40	92	23	11	76	49	6.129	0.0133
Vandevyver, 1997	Caucasian	Female	12	50	24	127	368	203	3.142	0.0763
Tamai, 1997	East Asian	Female	5	11	74	3	16	73	2.784	0.0952
Yanagi, 1996	East Asian	Female	2	7	57	5	7	11	2.767	0.0962
Houston, 1996	Caucasian	Female	8	19	17	9	19	16	0.571	0.4498

**Table 3:** Genotype frequencies of VDR *FokI* polymorphism in studies included in this meta-analysis

First author/year	Ethnicity	Gender	Case			Control			HWE	
			FF	Ff	ff	FF	Ff	ff	Chi-square test	*P*
Techapatiphandee, 2018	Southeast Asian	Female	31	46	28	41	73	18	2.613	0.106
Ahmad, 2018	Indian	Female	148	92	14	169	80	5	1.637	0.2008
Mohammadi, 2015	West Asian	Female	80	56	3	11	17	3	0.95	0.3298
Mohammadi, 2015	West Asian	Female	52	36	8	198	128	30	1.996	0.1577
Mohammadi, 2015	West Asian	Male	40	26	3	111	73	9	0.476	0.4903
Mohammadi, 2015	West Asian	Male	64	41	4	12	9	1	0.182	0.6698
González, 2013	Caucasian	Female	24	45	19	25	48	15	0.974	0.3238
Yasovanthi, 2011	Indian	Female	104	119	24	122	124	8	12.594	0.0004
Yasovanthi, 2011	Indian	Female	73	82	25	97	101	8	8.71	0.0032
Xing, 2011	East Asian	Female	11	14	7	8	35	27	0.443	0.5058
Mansour, 2010	African	Female	34	9	7	20	0	0	0	0
Durusu, 2010	Caucasian	Female	27	22	1	29	18	3	0.009	0.9259
Gu, 2010	East Asian	Female	6	18	9	40	84	24	3.266	0.0707
Gu, 2010	East Asian	Male	2	5	1	76	137	47	1.171	0.2791
Mencej-Bedrac, 2009	Caucasian	Female	88	108	44	105	97	26	0.249	0.6179
Pérez, 2008	Caucasian	Female	22	32	10	22	36	10	0.586	0.4438
Mitra, 2006	Indian	Female	38	42	39	46	33	18	6.444	0.0111
Zhang, 2006	East Asian	Male	4	13	9	28	28	10	0.458	0.4984
Lisker, 2003	Caucasian	Female	27	29	9	20	29	8	0.239	0.625
Zajickova, 2002	Caucasian	Female	26	28	11	7	21	5	2.54	0.111
Langdahl, 2000	Caucasian	Male	12	13	5	30	34	9	0.018	0.8943
Langdahl, 2000	Caucasian	Female	28	42	10	34	31	15	2.554	0.11
Choi, 2000	East Asian	Female	12	23	13	26	33	6	0.961	0.327
Lucotte, 1999	Caucasian	Female	45	69	10	40	52	13	0.386	0.5346
Gennari, 1999	Caucasian	Female	60	73	31	53	55	11	0.372	0.542

**Table 4:** Genotype frequencies of VDR *Cdx2* polymorphism in studies included in this meta-analysis

First author/year	Ethnicity	Gender	Case			Control			HWE	
			GG	GA	AA	GG	GA	AA	Chi-square test	*P*
Ziablitsev, 2015	Caucasian	Female	16	20	8	2	12	16	0.015	0.9009
Marozik, 2013	Caucasian	Female	41	13	0	53	24	0	2.624	0.1052
Gu, 2010	East Asian	Female	12	16	5	38	72	38	0.108	0.7423
Gu, 2010	East Asian	Male	4	3	1	81	116	63	2.78	0.0955
Mencej-Bedrac, 2009	Caucasian	Female	155	75	9	172	48	8	3.709	0.0541

### Meta-analysis results

**Figure 2:**
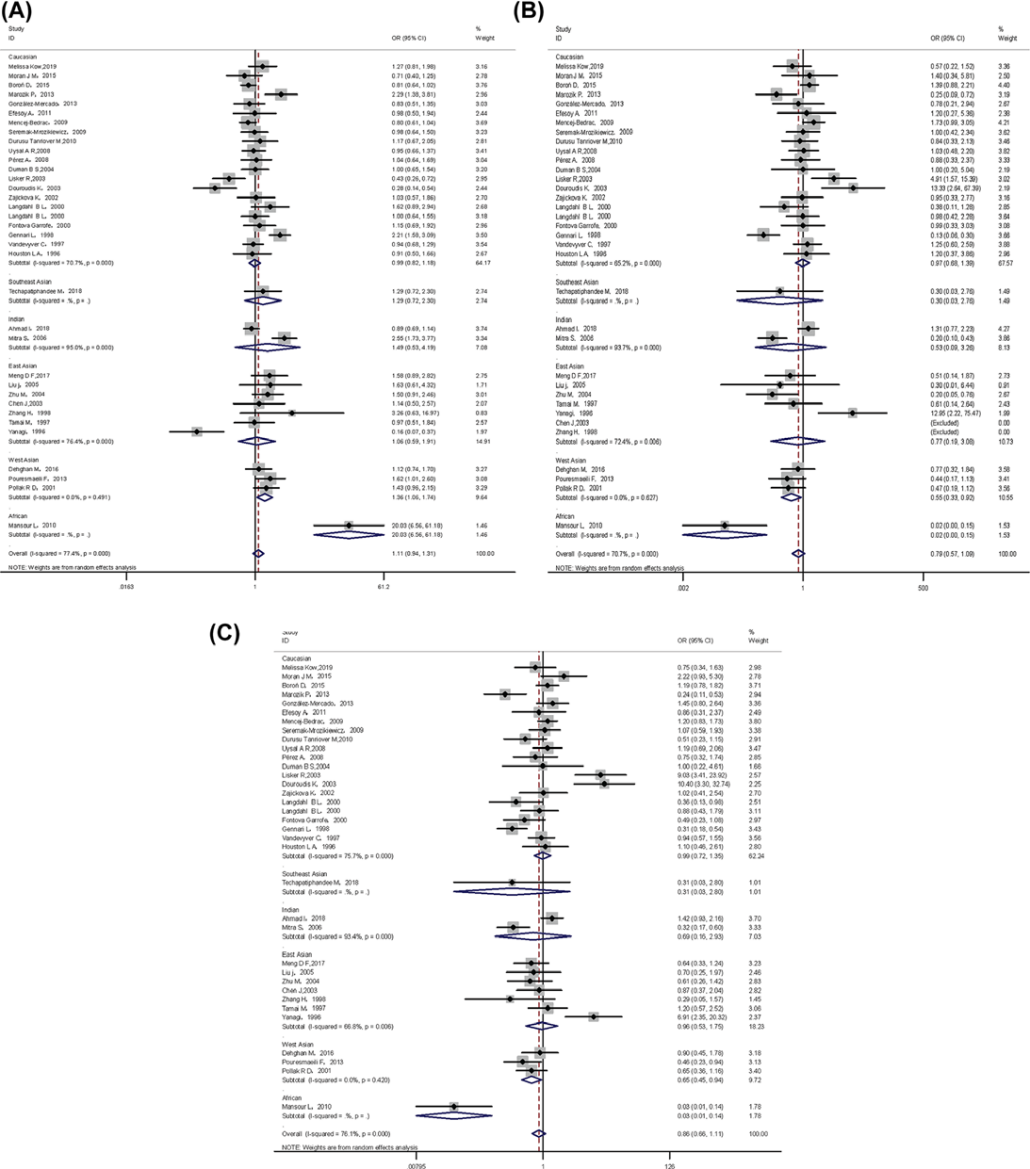
VDR BsmI polymorphism and osteoporosis risk in different races The forest plots of all selected studies on the association between VDR *BsmI* polymorphism and osteoporosis risk in different races (**A**) allele model; (**B**) additive model; (**C**) recessive model.

Table 5 summarizes the assessment of the association between *VDR* BsmI polymorphism and osteoporosis risk. Overall, significantly increased the risk of osteoporosis was not found for *VDR* BsmI polymorphism (*P*>0.05 in all genetic models). However, subgroup analysis by ethnicity, we observed that the *VDR* b allele genotype increased the osteoporosis risk (OR = 1.36, 95% CI: 1.06–1.74) and bb genotype (additive model: OR = 0.55, 95% CI: 0.33–0.92; recessive model: OR = 0.65, 95% CI: 0.45–0.96) reduced the risk of osteoporosis in the West Asians, as shown in Figure 2.

**Table 5:** Pooled estimates of association of VDR *BsmI* polymorphism and osteoporosis risk

Genetic model	Variable	Test of association		Tests for heterogeneity		Egger’s test
		OR (95% CI)	*P*	*P_h_*	*I^2^*	*P_E_*
B vs b	Overall	1.11 (0.94–1.31)	0.22	<0.001	77.40%	0.34
	Caucasian	0.99 (0.83–1.18)	0.87	<0.001	70.70%	
	East Asian	1.06 (0.59–1.91)	0.85	<0.001	76.40%	
	West Asian	1.36 (1.06–1.74)	0.02	0.49	0.00%	
	Indian	1.49 (0.53–4.19)	0.45	<0.001	95%	
	Female	1.09 (0.90–1.31)	0.39	<0.001	79.60%	
	Male	1.29 (0.99–1.67)	0.06	0.75	0.00%	
bb vs BB	Overall	0.79 (0.57–1.09)	0.15	<0.001	70.70%	0.28
	Caucasian	0.97 (0.68–1.39)	0.88	<0.001	65.20%	
	East Asian	0.77 (0.19–3.08)	0.71	0.01	72.40%	
	West Asian	0.55 (0.33–0.92)	0.02	0.63	0.00%	
	Indian	0.53 (0.09–3.26)	0.49	<0.001	93.70%	
	Female	0.82 (0.58–1.17)	0.28	<0.001	73.60%	
	Male	0.58 (0.33–1.02)	0.06	0.79	0.00%	
Bb+bb vs BB	Overall	0.87 (0.70-1.07)	0.19	<0.001	53.00%	0.15
	Caucasian	1.02 (0.83–1.27)	0.83	0.06	34.20%	
	East Asian	0.74 (0.22–2.46)	0.63	0.02	65.80%	
	West Asian	0.68 (0.44–1.07)	0.09	0.82	0.00%	
	Indian	0.58 (0.19–1.76)	0.34	<0.001	88.40%	
	Female	0.89 (0.70–1.12)	0.32	<0.001	57.70%	
	Male	0.71 (0.45–1.13)	0.15	0.94	0.00%	
bb vs BB+Bb	Overall	0.86 (0.67–1.11)	0.24	<0.001	76.10%	0.44
	Caucasian	0.99 (0.72–1.35)	0.94	<0.001	75.70%	
	East Asian	0.96 (0.53–1.75)	0.89	0.01	66.80%	
	West Asian	0.65 (0.45–0.96)	0.02	0.42	0.00%	
	Indian	0.69 (0.16–2.93)	0.61	<0.001	93.40%	
	Female	0.89 (0.67–1.17)	0.40	<0.001	78.30%	
	Male	0.70 (0.46–1.06)	0.09	0.53	0.00%	
BB+bb vs Bb	Overall	0.98 (0.82–1.15)	0.76	<0.001	55.20%	0.84
	Caucasian	0.98 (0.77–1.24)	0.85	<0.001	66.60%	
	East Asian	1.04 (0.68–1.59)	0.87	0.19	31.50%	
	West Asian	0.87 (0.61–1.22)	0.41	0.49	0.00%	
	Indian	1.19 (0.89–1.61)	0.24	0.51	0.00%	
	Female	0.98 (0.82–1.18)	0.86	<0.001	59.30%	
	Male	0.94 (0.65–1.35)	0.74	0.56	0.00%	

VDR *BsmI*: allele model: B vs b, additive model: bb vs BB, dominant model: Bb + bb vs BB, recessive model: bb vs BB + Bb, overdominance model: BB + bb vs Bb.

**Figure 3:**
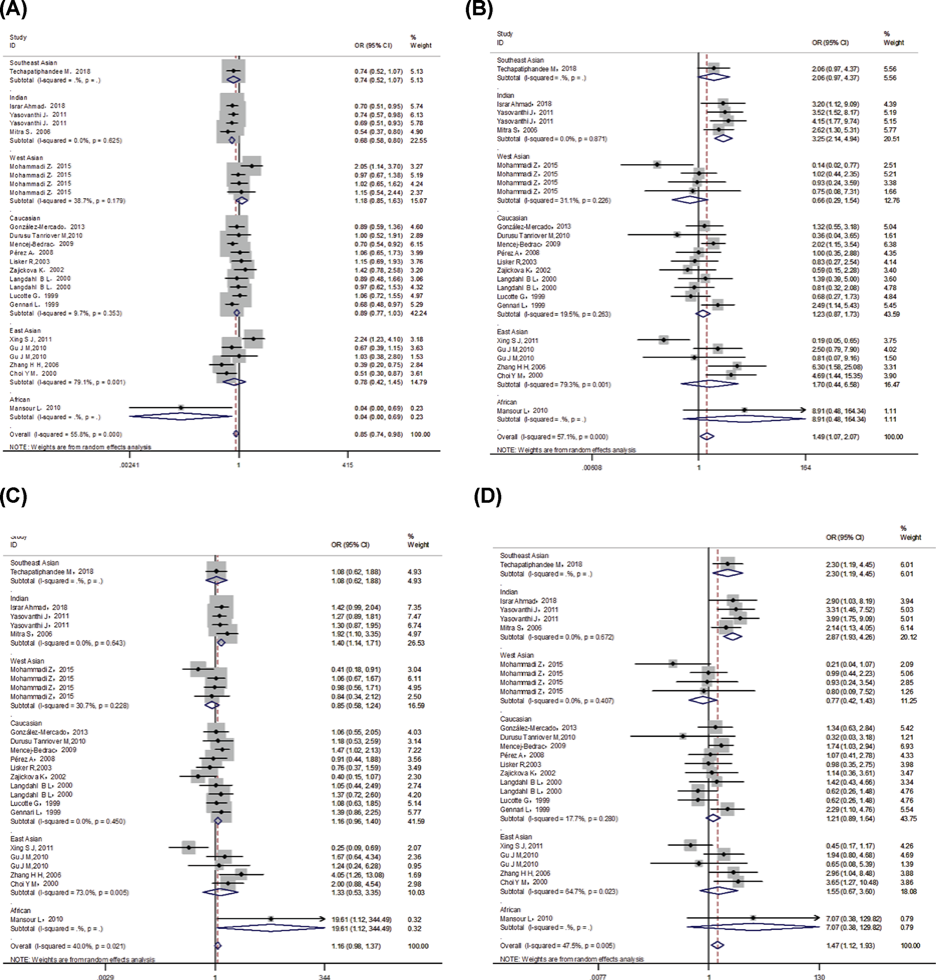
VDR FokI polymorphism and osteoporosis risk in different races The forest plots of all selected studies on the association between VDR *FokI* polymorphism and osteoporosis risk in different races (**A**) allele model; (**B**) additive model; (**C**) dominant model; (**D**) recessive model.

**Figure 4:**
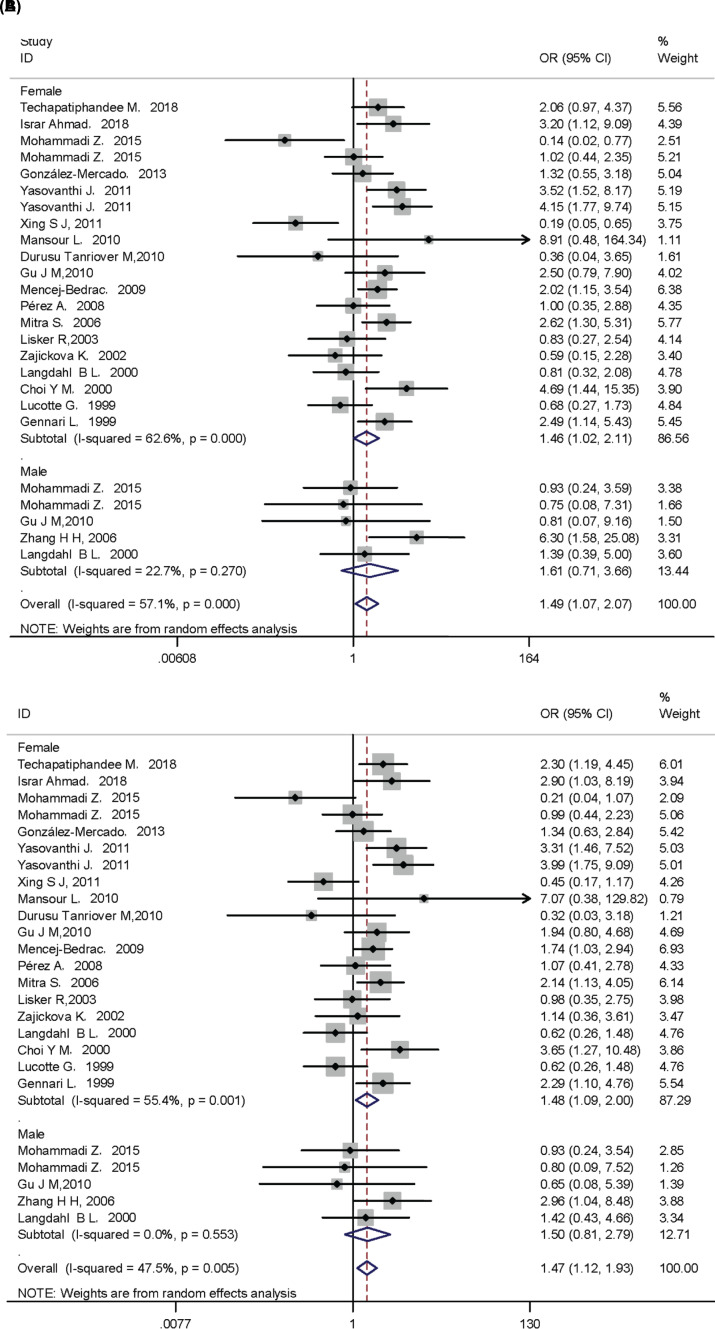
VDR FokI polymorphism and osteoporosis risk between different gender The forest plots of all selected studies on the association between VDR *FokI* polymorphism and osteoporosis risk between different gender (**A**) additive model; (**B**) recessive model.

At the overall analysis, significantly increased osteoporosis risk was found in *VDR* FokI ff genotype (additive model: OR = 1.49, 95% CI: 1.07–2.07; recessive model: OR = 1.47, 95% CI: 1.13–1.93). In addition, when stratified by ethnicity, the results showed that f allele and ff genotypes were significantly associated with risk of osteoporosis in Indians. We further performed subgroup analysis according to gender, significantly elevated osteoporosis risk was also observed in ff genotype. All the data are shown in Table 6, Figures 3 and 4.

**Table 6:** Pooled estimates of association of VDR *FokI* polymorphism and osteoporosis risk

Genetic model	Variable	Test of association		Tests for heterogeneity		Egger’s test
		OR (95% CI)	*P*	*P_h_*	*I^2^*	*P_E_*
F vs f	Overall	0.86 (0.74–0.98)	0.03	<0.001	55.80%	0.30
	Caucasian	0.89 (0.77–1.03)	0.12	0.35	9.70%	
	East Asian	0.78 (0.42–1.45)	0.43	0.001	79.10%	
	West Asian	1.18 (0.85–1.63)	0.32	0.002	73.90%	
	Indian	0.68 (0.58–0.80)	0	0.63	0.00%	
	Female	0.86 (0.74–1.00)	0.05	<0.001	59.90%	
	Male	0.83 (0.56–1.23)	0.35	0.14	41.90%	
ff vs FF	Overall	1.49 (1.07–2.07)	0.02	<0.001	57.10%	0.11
	Caucasian	1.23 (0.87–1.73)	0.24	0.26	19.50%	
	East Asian	1.69 (0.44–6.58)	0.45	0.001	79.30%	
	West Asian	0.66 (0.29–1.54)	0.34	0.23	31.10%	
	Indian	3.25 (2.14–4.94)	0	0.87	0.00%	
	Female	1.46 (1.02–2.11)	0.04	<0.001	62.60%	
	Male	1.61 (0.71–3.66)	0.25	0.27	22.70%	
Ff+ff vs FF	Overall	1.16 (0.98–1.37)	0.08	0.02	40.00%	0.42
	Caucasian	1.16 (0.96–1.40)	0.12	0.45	0.00%	
	East Asian	1.33 (0.53–3.35)	0.55	0.01	73.00%	
	West Asian	0.85 (0.58–1.24)	0.40	0.23	30.70%	
	Indian	1.40 (1.14–1.71)	0.001	0.64	0.00%	
	Female	1.15 (0.96–1.38)	0.12	0.02	45.20%	
	Male	1.19 (0.74–1.90)	0.47	0.26	24.10%	
ff vs FF+Ff	Overall	1.47 (1.13–1.93)	0.01	0.01	47.50%	0.13
	Caucasian	1.21 (0.89–1.64)	0.24	0.28	17.70%	
	East Asian	1.55 (0.67–3.60)	0.31	0.02	64.70%	
	West Asian	0.77 (0.42–1.43)	0.41	0.41	0.00%	
	Indian	2.87 (1.93–4.26)	0	0.67	0.00%	
	Female	1.48 (1.09–2.00)	0.01	0.001	55.40%	
	Male	1.50 (0.81–2.79)	0.20	0.55	0.00%	
FF+ff vs Ff	Overall	1.01 (0.90–1.13)	0.87	0.69	0.00%	0.96
	Caucasian	0.97 (0.81–1.18)	0.78	0.41	3.60%	
	East Asian	1.02 (0.69–1.51)	0.91	0.88	0.00%	
	West Asian	1.06 (0.78–1.45)	0.71	0.53	0.00%	
	Indian	0.97 (0.80–1.19)	0.80	0.63	0.00%	
	Female	1.03 (0.90–1.15)	0.78	0.45	0.80%	
	Male	0.94 (0.65–1.37)	0.76	0.93	0.00%	

VDR *FokI*: allele model: F vs f, additive model: ff vs FF, dominant model: Ff+ff vs FF, recessive model: ff vs FF+Ff, overdominance model: FF+ff vs Ff.

No significant association was observed between *VDR* Cdx2 polymorphism and osteoporosis risk, as shown in Table 7.

**Table 7:** Pooled estimates of association of VDR *Cdx2* polymorphism and osteoporosis risk

Genetic model	Test of association		Tests for heterogeneity		Egger’s test
	OR (95% CI)	*P*	*P_h_*	*I^2^*	*P_E_*
G vs A	1.54 (0.80–2.97)	0.20	<0.001	82.40%	0.12
AA VS GG	0.37 (0.11–1.28)	0.11	0.02	68.30%	0.29
GA+AA VS GG	0.64 (0.29–0.39)	0.27	0.002	75.70%	0.01
AA VS GG+GA	0.48 (0.22–1.07)	0.07	0.14	45.70%	0.85
GG+AA VS GA	0.84 (0.58–1.22)	0.36	0.28	21.30%	0.12

VDR *Cdx2*: allele model: G vs A, additive model: AA VS GG, dominant model: GA+AA VS GG, recessive model: AA VS GG+GA, overdominance model: GG+AA VS GA.

### Heterogeneity and sensitivity analyses

Heterogeneity was observed in overall and several subgroup analyses. Some potential factors were considered as sources of heterogeneity, such as ethnicity, gender, HWE, and menopausal status. Then, we applied meta-regression analysis to explore sources of heterogeneity. The results suggested that the studies of HWD were source of heterogeneity in overall population (additive model: *P*=0.024). In addition, the studies of HWD was also the source of heterogeneity on the association between women and osteoporosis risk (additive model: *P*=0.029 and recessive model: *P*=0.025).

Sensitivity analysis was estimated by applying three methods in this meta-analysis. First, results did not change when removing a single study each time to appraise the robustness. However, when we excluded studies of low quality and HWD, significantly decreased osteoporosis risk was found in overall analysis for *VDR* BsmI bb genotype (additive model: OR = 0.74, 95% CI: 0.56–0.99; recessive model: OR = 0.79, 95% CI: 0.63–0.98). Further, when we restrained only including high-quality HWE, and matching studies, the corresponding pooled OR do not appear to be significantly affected. Therefore, the results of the sensitivity analysis are shown in Tables 8 and 9.

**Table 8:** Pooled estimates of association of VDR *BsmI, FokI, Cdx2* polymorphism and osteoporosis risk, excluding low quality and HWD studies

Genetic model	Test of association		Tests for heterogeneity	
	OR (95% CI)	*P*	*P_h_*	*I^2^*
VDR *BsmI*				
B vs b	1.16 (1.00–1.35)	0.05	0.002	53.00%
bb vs BB	0.74 (0.56–0.99)	0.04	0.021	42.50%
Bb+bb vs BB	0.88 (0.72–1.08)	0.22	0.194	20.60%
bb vs BB+Bb	0.79 (0.63–0.98)	0.04	0.004	50.70%
BB+bb vs Bb	0.91 (0.79–1.06)	0.23	0.224	17.80%
VDR *FokI*				
F vs f	0.93 (0.81–1.08)	0.33	0.009	48.00%
ff VS FF	1.17 (0.83–1.66)	0.37	0.006	50.20%
Ff+ff VS FF	1.07 (0.89–1.27)	0.47	0.080	32.60%
ff VS FF+Ff	1.23 (0.93–1.63)	0.16	0.036	39.60%
FF+ff VS Ff	1.01 (0.88–1.15)	0.90	0.596	0.00%
VDR *Cdx2*				
G vs A	1.17 (0.68–2.00)	0.57	0.026	67.50%
AA VS GG	0.68 (0.29–1.58)	0.37	0.269	23.80%
GA+AA VS GG	0.86 (0.44–1.66)	0.65	0.030	66.40%
AA VS GG+GA	0.72 (0.37–1.40)	0.34	0.531	0.00%
GG+AA VS GA	0.89 (0.55–1.45)	0.64	0.166	41.00%

**Table 9:** Pooled estimates of association of VDR *BsmI, FokI* polymorphism and osteoporosis risk, only studies with high-quality matching, and studies conforming to HWE

Genetic model	Test of association		Test for heterogeneity	
	OR (95% CI)	*P*	*P_h_*	*I^2^*
VDR *BsmI*				
B vs b	1.14 (0.96–1.36)	0.14	0.469	0.00%
bb VS BB	0.71 (0.48–1.03)	0.07	0.652	0.00%
Bb+bb VS BB	0.86 (0.64–1.14)	0.28	0.870	0.00%
bb VS BB+Bb	0.81 (0.61–1.08)	0.15	0.215	26.80%
BB+bb VS Bb	0.96 (0.76–1.22)	0.74	0.410	2.60%
VDR *FokI*				
F vs f	0.96 (0.81–1.14)	0.63	0.157	31.50%
ff VS FF	1.17 (0.84–1.61)	0.36	0.120	36.00%
Ff+ff VS FF	1.08 (0.91–1.30)	0.39	0.434	0.40%
ff VS FF+Ff	1.16 (0.86–1.57)	0.35	0.069	43.30%
FF+ff VS Ff	0.97 (0.81–1.15)	0.70	0.301	15.50%

### Publication bias

**Figure 5:**
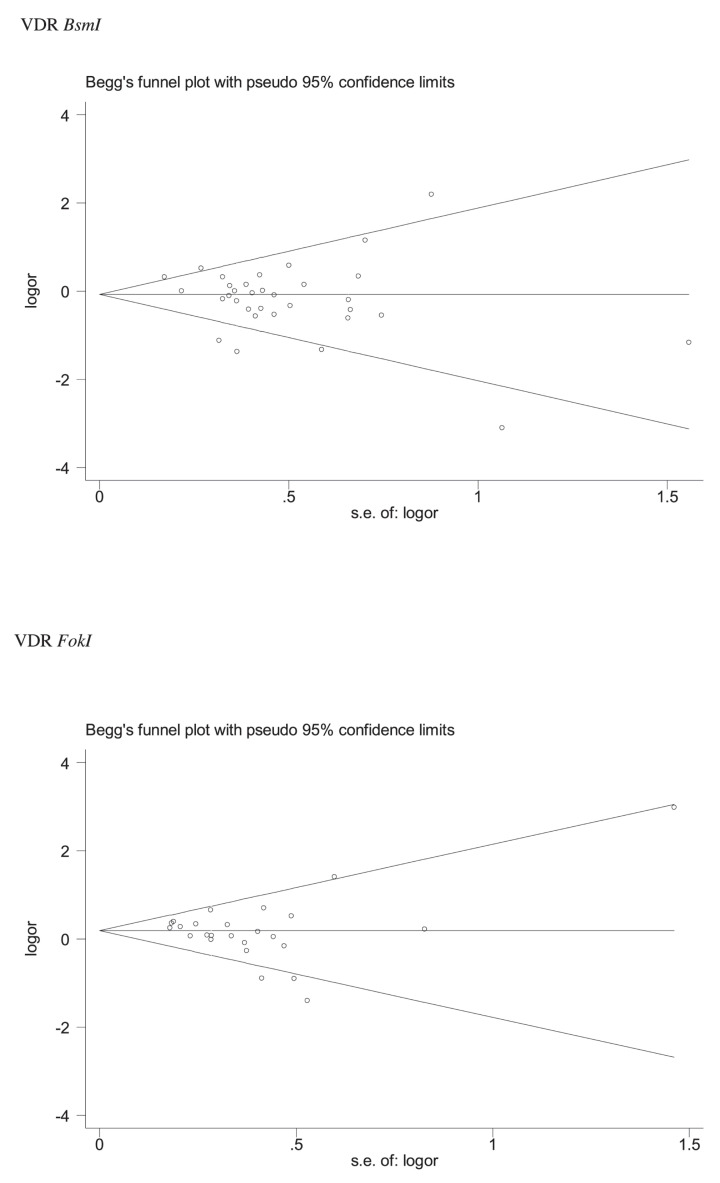
Begg’s funnel plot to assess publication bias

Publication bias was assessed in the overall publication by Begg’s funnel plot and Egger’s test, the shape of the funnel plots revealed no significant funnel asymmetry (Figure 5) in overall population. The Egger tests also indicated that there was no obvious evidence of publication bias (*P*>0.05 in all genetic models), as shown in Tables 5–7.

### Credibility of the identified genetic associations

We classified statistically significant associations that met the following criteria as ‘positive results’ [81]: (1) the *P*-value of Z-test is less than 0.05 in at least two gene models; (2) at the *P*-value level of 0.05, the FPRP is less than 0.2; (3) statistical power > 0.8; (4) *I^2^* < 50%. Considered as ‘less credible affirmation’ with lower threshold when the following conditions were met: (1) *P*-value <0.05 in at least one of the genetic models; (2) the statistical power was between 50 and 79% or FPRP > 0.2 or *I^2^* > 50%. Otherwise, the association was classified as ‘null’ or ‘negative’. After credibility assessment, we identified ‘less-credible positive results’ for the statistically significant associations in the current meta-analysis. The detailed credibility assessment results are listed in Table 10.

**Table 10:** FPRP values for the statistically significant associations in current meta-analysis

Variables	OR (95% CI)	*I^2^* (%)	Statistical power		Prior probability of 0.001	
			OR = 1.2	OR = 1.5	OR = 1.2	OR = 1.5
Overall						
ff vs FF	1.49 (1.07–2.07)	57.10%	0.098	0.516	0.994	0.971
ff vs FF+Ff	1.47 (1.13–1.93)	47.50%	0.072	0.558	0.987	0.909
West Asian						
B vs b	1.36 (1.06–1.74)	0%	0.160	0.782	0.989	0.949
bb vs BB	0.55 (0.33–0.92)	0%	0.057	0.232	0.998	0.990
bb vs BB+Bb	0.65 (0.45–0.96)	0%	0.106	0.449	0.997	0.985
Indian						
F vs f	0.68 (0.58–0.80)	0%	0.007	0.594	0.317	0.006
ff vs FF	3.25 (2.14–4.94)	0%	0	0	0.957	0.189
Ff+ff vs FF	1.40 (1.14–1.71)	0%	0.065	0.75	0.937	0.565
ff vs FF+Ff	2.87 (1.93–4.26)	0%	0	0.001	0.957	0.207
Female						
ff vs FF	1.46 (1.02–2.11)	62.60%	0.148	0.557	0.997	0.987
ff vs FF+Ff	1.48 (1.09–2.00)	55.40%	0.086	0.535	0.992	0.952
Exclude low quality and HWD studies						
Overall						
bb VS BB	0.74 (0.56–0.99)	42.50%	0.212	0.759	0.995	0.982
bb VS BB+Bb	0.79 (0.63–0.98)	50.70%	0.314	0.939	0.99	0.972

## Discussion

Osteoporosis is a multifactorial disease and is strongly related to heredity [7]. Genes are very important factors for the risk of osteoporosis. Osteoporosis is characterized by low BMD and microarchitectural deterioration of bone leading to increased bone fragility and a high risk of fracture. The *VDR* gene is considered as a candidate gene and has been widely studied due to it plays a key role in regulating bone resorption and metabolism [10]. And the VDR gene has also been implicated as a factor affecting bone mass [84]. Hence, it will be very important to investigate the association between *VDR* gene polymorphism and osteoporosis. Moreover, the VDR polymorphisms play an important role in the pathogenesis, prevention, diagnosis and treatment of osteoporosis and other disease such as acute ischemic stroke [85]. In addition, single nucleotide polymorphism (SNP) may affect the function of *VDR* and may be related with osteoporosis risk [82]. Although many studies attempted to explore the association between *VDR* polymorphisms and the risk of osteoporosis. However, it is regrettable that no solid evidence has been obtained, which may be due to different reasons, including small sample size, ethnic, and regional differences. In order to overcome these shortcomings, meta-analysis is effective alternative.

A total of six previous meta-analyses explored the association between *VDR* polymorphisms and osteoporosis risk. Wang et al. [24] and Yu et al. [26] explored the association between osteoporosis risk and *VDR* BsmI polymorphism in Chinese and Han Chinese population, respectively. Their results suggested that there was no significant association between *VDR* BsmI polymorphism and osteoporosis risk. In 2013, Jia et al. [27] examined 26 studies including 2274 cases and 3150 controls to show that the *VDR* BsmI polymorphism was associated with an decreased osteoporosis risk. However, the examination of 41 studies on *VDR* BsmI polymorphism (including 3080 cases and 4157 controls) by Gang et al. [28] indicated that the *VDR* BsmI polymorphism was not significantly associated with osteoporosis risk. In addition, the examination of 36 studies on *VDR* BsmI, 15 studies on *VDR* FokI, and three studies on *VDR* Cdx2 by Zhang et al. [25] indicated that the *VDR* BsmI and *VDR* FokI polymorphisms were associated with an increased the risk of developing osteoporosis in overall and Asians, while the *VDR* Cdx2 polymorphism may be not associated with osteoporosis risk. However, *VDR* BsmI and *VDR* FokI polymorphisms had not been found to increase the risk of osteoporosis by Zintzaras et al. [29]. Further, when we examined these meta-analyses carefully, we found some disadvantages. First, quality assessments of the eligible studies had not been performed in some studies [24,25,27–29], and low-quality literature may be included in these meta-analyses, resulting in deviation of the results. Second, HWE is absolutely necessary for a sound genetic association study. There may be selection bias or genotyping errors if the control group did not meet HWE. It can lead to misleading results. The distribution of genotypes in the control group was not tested by HWE [24,25]. Then, the statistical power was not calculated in some previous meta-analyses [24,26–29]. Finally, the FPRPs of statistically significant association was not evaluated in all previous meta-analyses [24–29]. Therefore, results of their meta-analyses may be not credible.

A total of 43 studies were included in the current meta-analysis, of which 34 studies explored the association between *VDR* BsmI and osteoporosis risk, 19 studies reported *VDR* FokI polymorphism, and four studies related to *VDR* Cdx2 polymorphism. Furthermore, five genetic models are compared separately. Overall, compared with the FF and Ff genotypes, statistically significant increased osteoporosis risk was found in the *VDR* FokI ff genotype. In the subgroup analysis, the *VDR* FokI ff genotype was significantly associated with increased osteoporosis risk in Indians and women population. However, significantly decreased the risk of osteoporosis were observed in the West Asians for *VDR* BsmI b allele and bb genotype. In addition, when we excluded studies of low quality and HWD, a significantly decreased the risk of osteoporosis was found in the overall analysis for the *VDR* BsmI bb genotype. Further, significant association did not observed when the pooled analysis was limited only involving high quality, HWE, and matching studies. Furthermore, the current meta-analysis was performed by applying multiple subgroups and different genetic models, at the cost of multiple comparisons, in which case the pooled *P*-value must be adjusted [83]. The Venice criteria, statistical power, and *I*^2^ value were very important criteria [37]. Hence, the FPRP test and Venice criteria were used to assess positive results. After credibility assessment, we identified ‘less-credible positive results’ for the statistically significant associations in the current meta-analysis. Heterogeneity has also been observed in the current meta-analysis. Results of meta-regression analysis suggested that studies of HWD were the source of heterogeneity. In addition, no obvious asymmetry was found in the study of VDR *BsmI* and *FokI* by the Begg’s funnel plots and Egger tests. Due to the limited number of studies, the Begg’s funnel plot was not performed to explored publication bias in the VDR *Cdx2* study. Meantime, the Egger tests revealed that there was no clear statistical evidence of publication bias.

The current meta-analysis has the following advantages: (1) the quality of included studies was assessed; (2) the HWE test was performed in the control group; (3) we applied FPRP and Venice criteria to evaluate the significant association in current meta-analysis; (4) the sample size was much larger than the previous meta-analyses; (5) we explored sources of heterogeneity based on meta-regression analysis. However, there are still some limitations in the present study. First, we did not control confounding factors such as smoking, drinking, and variable study designs, were closely related to affect the results. Second, in the subgroup analyses, the number of studies were relatively small in Indians, and there was not enough statistical power to explore the real association. Moreover, due to the limited number of studies, we did not perform subgroup analyses in the pooled analysis of *VDR* Cdx2 polymorphism and osteoporosis risk. Therefore, the study with large sample size and large enough subgroup will help to verify our findings.

In conclusion, these positive findings should be interpreted with caution and indicate that significant association may most likely result from less-credible, rather than from true associations or biological factors on the *VDR* BsmI and FokI polymorphisms with osteoporosis risk*.*

## Supplementary Material

Supplementary Table S1

## Abbreviations

BMD: bone mineral density
FPRP: false-positive report probability
HWD: Hardy–Weinberg disequilibrium
HWE: Hardy–Weinberg equilibrium
LS: lumbar spine
OR: odds ratio
SNP: single nucleotide polymorphism
VDR: vitamin D receptor
95% CI: 95% confidence interval
