# Supplementary material for: Association between vitamin D receptor BsmI, FokI, and Cdx2 polymorphisms and osteoporosis risk: an updated meta-analysis
Source: Biosci Rep. 2020 Jul 15;40(7):BSR20201200. doi: 10.1042/BSR20201200 (PMC7364509; doi:10.1042/BSR20201200)
Supplement: Supplementary Table S1 [file BSR-2020-1200_supp.pdf]

Supplementary Table 1. Scale for quality assessment of molecular association studies

Criterion	Score
Source of case	
Selected from population	2
Selected from hospital	1
Not described	0
Source of control	
Population-based	3
Blood donors or volunteers	2
Hospital-based	1
Not described	0
Ascertainment of osteoporosis	
WHO	**2**
Diagnosis of osteoporosis by patient medical record	1
Not described	0
Ascertainment of control	
Controls were tested to screen out	2
Controls were subjects who did not report osteoporosis, no objective testing	1
Not described	0
Matching	
Controls matched with cases by age and sex	2
Controls matched with cases only by age or sex	1
Not matched or not described	0
Genotyping examination	
Genotyping done blindly and quality control	2
Only genotyping done blindly or quality control	1
Unblinded and without quality control	0
Specimens used for determining genotypes	
Blood cells or normal tissues	1
Tumor tissues or exfoliated cells of tissue	0
HWE	
HWE in the control group	1
Hardy-Weinberg disequilibrium in the control group	0
Association assessment	
Assess association between genotypes and osteoporosis with appropriate statistics and adjustment for confounders	2
Assess association between genotypes and osteoporosis with appropriate statistics without adjustment for confounders	1
Inappropriate statistics used	0
Total sample size	
>500	3
200-500	2
<200	1

HWE: Hardy-Weinberg equilibrium
